# Lead-free 2D MASnBr_3_ and Ruddlesden–Popper BA_2_MASn_2_Br_7_ as light harvesting materials†

**DOI:** 10.1039/d3ra00108c

**Published:** 2023-03-09

**Authors:** Sandip R. Kumavat, Yogesh Sonvane

**Affiliations:** a Advanced Materials Lab, Department of Physics, Sardar Vallabhbhai National Institute of Technology Surat 395007 India yas@phy.svnit.ac.in

## Abstract

We have explored the structural, electronic, charge transport, and optical properties of lead-free 2D hybrid halide perovskites, MASnBr_3_ and Ruddlesden–Popper perovskites, BAMASn_2_Br_7_ monolayers. Under density functional theory (DFT) calculation, we applied mechanical strain, *i.e.*, tensile and compressive strain up to 10% in both cases. The mechanical strain engineering technique is useful for a tuned bandgap of 2D MASnBr_3_ and 2D BAMASn_2_Br_7_. The calculated carrier mobility for the electron is 404 cm^2^ V^−1^ s^−1^ and for the hole is up to 800 cm^2^ V^−1^ s^−1^ for MASnBr_3_. For BAMASn_2_Br_7_ the highest carrier mobility is up to 557 cm^2^ V^−1^ s^−1^ for electrons and up to 779 cm^2^ V^−1^ s^−1^ for the hole, which is 14% and 24% higher than the reported lead-iodide based perovskites, respectively. The calculated solar cell efficiency of 2D MASnBr_3_ is 23.46%, which is 18% higher than the reported lead-based perovskites. Furthermore, the optical activity of the 2D MASnBr_3_ and 2D BAMASn_2_Br_7_ shows a high static dielectric constant of 2.48 and 2.14, respectively. This is useful to show nanodevice performance. Also, 2D MASNBr_3_ shows a high absorption coefficient of 15.25 × 10^5^ cm^−1^ and 2D BAMASn_2_Br_7_ shows an absorption coefficient of up to 13.38 × 10^5^ cm^−1^. Therefore our theoretical results suggest that the systems are under mechanical strain engineering. This is convenient for experimentalists to improve the performance of the 2D perovskites. The study supports these materials as good candidates for photovoltaic and optoelectronic device applications.

## Abstract 2

The calculated band gap of (a) MASnBr_3_ (b) BAMASn_2_Br_7_ with strain engineering for 0%, 1% , 2%, 4%, 6%, 8% and 10% respectively.

## Graphical abstract

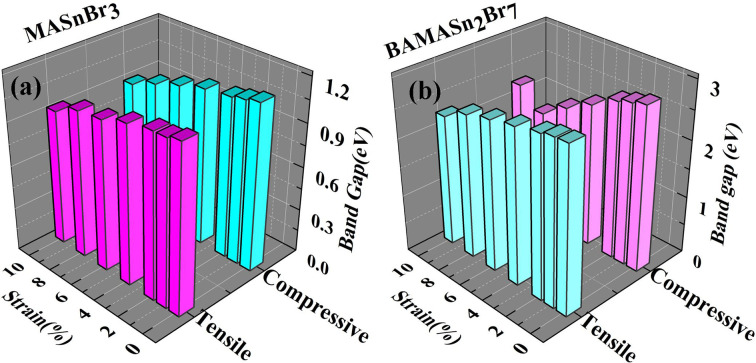

## 1. Introduction

Nowadays, energy crises and environmental pollution are the main big problems for humans. Many research groups are working on finding alternative solutions for these problems. Globally, educational and industrial purpose scientific communities are now attracted to renewable energy sources, and have developed new technology to overcome such situations and find low-cost solutions. The research community has recently become interested in organic–inorganic hybrid halide perovskites because of their rapid advancement in photovoltaics and optoelectronics over the past ten years.^1^ Compared with traditional silicon cells, these hybrid halide perovskites solar cells reached a power conversion efficiency (PCE) of more than 24% within a few years.^1–4^ Their electronic properties include a high extinction coefficient, tunable bandgaps, long carrier mobility, and a long charge transport diffusion path.^5–8^ However, with these advantages, chemical instability is the central issue. These hybrid halide perovskites being highly reactive with moisture is the main drawback for industrial purposes.^9–12^

Many technologies have developed and extracted the two-dimensional 2D slab by slicing from the 3D framework of hybrid halide perovskites.^13–15^ The transformation of 3D to 2D perovskite opens the potential to more clearly analyze electronic, optical, and transport properties.^16^ These 2D hybrid halide perovskites are a stable candidate for optoelectronic devices. Like light-emitting diodes,^17^ photocatalysts, field-effect transistors,^17–19^ lasers, *etc.*^20–25^ Also, these 2D perovskites have flexible structural and compositional properties. Long-term stability characteristics in ambient conditions, more moisture resistance than 3D structure, and tunable photovoltaic properties.^26–30^ 2D third-generation hybrid halide perovskites have the structural formula MAZX_3_ (ref. 31–34) (MA = CH_3_NH_3_, Z = Pb, Sn and X = Cl, Br, I). These 2D perovskites interact well with inorganic layers, same as Ruddlesden–Popper 2D structures,^35–40^ which has structural formula (BA)_2_MAZ_2_X_7_ (BA = CH_3_(CH_2_)_3_NH_3_, MA = CH_3_NH_3_, Z = Pb, Sn, X = Cl, Br, I). Due to the structural flexibility of 2D perovskites, many groups have developed a new technique to find its efficient power conversion efficiency (PCE). 2D material interfacing with 2D perovskites has reached PCE 12.6%.^12,15,41^ 2D lead halide CH_3_NH_3_PbI_3_ and Ruddlesden–Popper (CH_3_(CH_2_)_3_NH_3_)_2_CH_3_NH_3_Pb_2_I_7_ have reached 14.9%, 17%, and 19.7% PCE in theoretical experiments, respectively.^42–44^ Yang *et al.*'s group reached 20% PCE by interfacing 2D with 3D perovskites.^45^ Under condition Shockley–Queisser limit, PCE of perovskites was achieved 31% with the theoretical study.^46^ The 2D lead (Pb) hybrid halide perovskites have been considered good candidates as light-harvesting materials. But has toxicity issues with environmental effects.^47^ To solve the problem of environmentally and humanly hazardous hybrid halide perovskites based on lead (Pb). Researchers have discovered that substituting alternative divalent elements, such as In, Sn, Sb, Bi, *etc.*, for lead is advantageous for both the environment and human beings. Experimentally, the tin (Sn) based halide perovskites are isostructural compounds of Pb-based halide.^31^ Sn-based halide perovskites materials contain a narrow optical bandgap high optical absorption coefficient. Also, it contains high charge carrier mobility with low exiting binding energy and good stability.^48–50^ Experimentally, the tin-based halide materials work as a light-harvesting material covering a wide range of visible spectrums. Furthermore, we have found that many groups are working on lead-free tin-based hybrid halide perovskites. The Xu *et al.* group is tuning the optical band gap of perovskites for light harvester materials.^51^ For thermoelectric devices, these materials were used.^52^ Ruddlesden–Popper nonlinear band behaviour due to mismatch s-states of Pb and Sn design a metal alloy.^36^ As the number of 2D layers increases band, behaviour shows charge carries effect well compared with experimental result.^53^ Therefore, these studies are useful for developing new building blocks for future photovoltaic and optoelectronic devices.^54–62^

In this paper, we give a comparative analysis of two orthorhombic structures of hybrid halide perovskites using density functional theory (DFT) calculations. CH_3_NH_3_SnBr_3_ as (MASnBr_3_) (MA = CH_3_NH_3_) and Ruddlesden–Popper (CH_3_(CH_2_)_3_NH_3_)_2_CH_3_NH_3_Sn_2_Br_7_ as (BAMASn_2_Br_7_) (BA = (CH_3_(CH_2_)_3_NH_3_)_2_, MA = CH_3_NH_3_) perovskites with their structural, electronic, optical and transport properties. Under mechanical strain conditions, we apply both tensile and compressive strain up to 10%. Mechanical strain condition is a valuable way to tune the electronic and optical properties of 2D halide perovskites because of the flexible structural and composition properties. For the creation of lead-free 2D hybrid halide perovskites for optoelectronic and photovoltaic applications, mechanical strain engineering offers a novel route.

## 2. Methodology

Density functional theory (DFT) and the Vienna *ab initio* simulation package (VASP) are used for all structural and electrical calculations.^63–66^ The projected augmented wave (PAW) method used the generalized gradient approximation (GGA) exchange–correlation Perdew–Burke–Ernzerhof (PBE) functional.^66^ The van der Waals (vdW) is included in our calculations. We also include SOC and HSE calculation to find more clear results.^67^ The plane-wave kinetic energy cut-off is set to 500 eV. The Brillouin zone (BZ) *k*-point grid (5 × 5 × 1) was used for structure optimisation. Also (15 × 15 × 1) *k*-point grid was selected for further electronic properties. All atomic structures of MASnBr_3_ (MA = CH_3_NH_3_) and Ruddlesden–Popper (BA)_2_MASn_2_Br_7_ (BA = CH_3_(CH_2_)_3_NH_3_, MA = CH_3_NH_3_) are converged up to the force 0.0001 eV Å^−1^ and total energy at 10^−4^ eV. To define structural balance, we used the ionic radii based tolerance factor formula *t* = (*R*_A_ + *R*_X_)/√2(*R*_B_ + *R*_X_) should be in the range between 0.813 and 1.107.^5^ Optical properties can be estimated through the Kramers–Kronig transformation based on the electronic structure.^68–71^ The optical absorption coefficient can be calculated using the formula:1
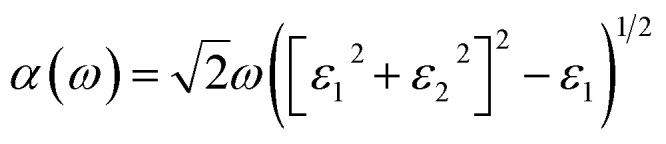


## 3. Results and discussions

### 3.1 Structural and electronic properties

**Fig. 1:**
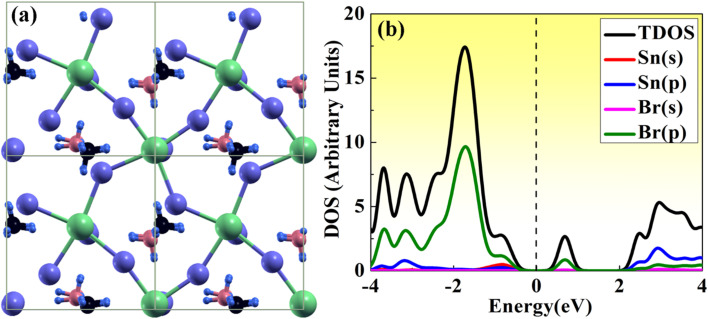
(a) Atomic structure of MASnBr_3_, where Sn, Br, C, N, H atom are represented in green, violet, black, dark pink, sky blue respectively. (b) Calculated total (TDOS) and projected density of states (PDOS) of MASnBr_3_.

**Fig. 2:**
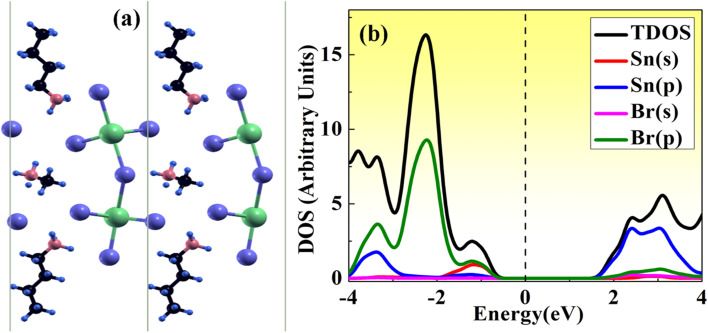
(a) Atomic structure of BAMASn_2_Br_7_, where Sn, Br, C, N, H atom are represented in green, violet, black, dark pink, sky blue respectively. (b) Calculated total (TDOS) and projected density of states (PDOS) of BAMASn_2_Br_7_.

Here, we have studied the structural parameter of hybrid halide MASnBr_3_ and BAMASn_2_Br_7_ monolayer, as shown in Fig. 1(a) and 2(a). The lattice parameters exhibit good agreement with those that have been previously reported, as shown in Table 1. We have not found much research on BAMASn_2_Br_7_ monolayers during the literature survey. Here we replaced lead (Pb) with tin (Sn),^41,43^ which has an ionic radius smaller than Pb and the ionic radius of halogen iodide (I) replaced by bromine (Br). The corresponding lattice parameters are of the 2D MASnBr_3,_ and BAMASn_2_Br_7_ monolayer smaller respectively as lead based 2D perovskites.^10,41,43^

**Table 1:** Calculated lattice parameter of MASnBr_3_ and BAMASn_2_Br_7_ monolayer

	*a* (Å)	*b* (Å)	Other
MASnBr_3_	8.08	8.5	8.5 (ref. 54 and 55)
BAMASn_2_Br_7_	7.20	5.21	6.0 (ref. 57)

With all these replacements, Sn and Br perovskites structure becomes more stable and does not have much change in the structural phase. Also, for both structures corresponding tolerance factor is in the range of 0.8 < *t* < 1.0, with consistence formation energy 141 eV for MASnBr_3_ and 196 eV for BAMASn_2_Br_7_.

**Fig. 3:**
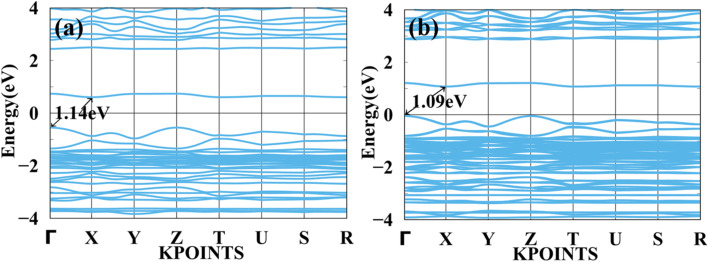
Calculated band structure of MASnBr_3_ with (a) PBE and (b) spin orbit coupling (SOC) represented.

**Fig. 4:**
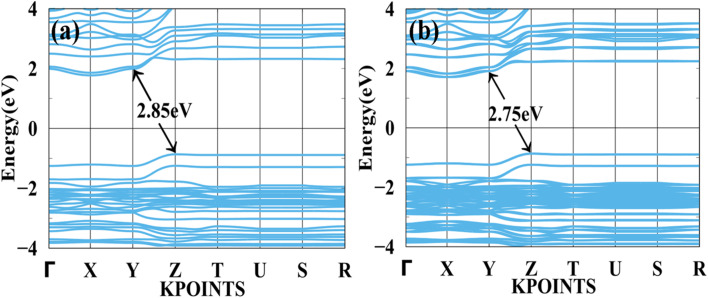
Calculated band structure of BAMASn_2_Br_7_ with (a) PBE and (b) spin orbit coupling (SOC) represented.

Furthermore, we have calculated the total density of states (TDOS) and projected density of states (PDOS) of MASnBr_3_ and BAMASn_2_Br_7_ in Fig. 1(b) and 2(b). In Fig. 1(b) it is clearly seen that in the valence band of MASnBr_3_, most of the part is contributed with the Br-p states with Sn-s states. While in the conduction band, most of the part is covered by the Sn-p states. Similarly, in the case of the Ruddlesden–Popper BAMASn_2_Br_7_ perovskites, most of the part valence band is contributed with the Sn-s and Br-p states. The conduction band is coved with the Sn-p states as shown in Fig. 2(b). Also, in both systems, null contributions of organic molecules in valence and conduction bands are observed. In Fig. 3 and 4 shows the calculated band structure of the MASnBr_3_ and BAMASn_2_Br_7_. Fig. 3(a) contain the band structure of MASnBr_3_ with PBE (without SOC) and Fig. 3(b) with SOC. The calculated indirect bandgap is along with gamma to *x* point 1.14 eV. Which is consistent with previously reported results.^52^

In Fig. 4(a) shows a bandgap of BAMASn_2_Br_7_ PBE (without SOC) and Fig. 4(b) with SOC. Here, the case of Ruddlesden–Popper perovskite BAMASn_2_Br_7_ indirect bandgap is at *Z* to *Y* point 2.85 eV. BAMASn_2_Br_7_ perovskites have not been much studied, but compared with related Ruddlesden–Popper are like BAMASn_2_I_7_ consistent.^36,43,58,59^ Also, when we replace Pb with Sn and iodine by Br, corresponding lattice parameters decrease with increasing corresponding band gaps.^41^ In both cases, we have seen that under the SOC study, there is not much effective change in indirect bandgap observed.^5,41,43^ An orbital contribution point of view in valence band maxima (VBM) is mostly contributed by the Sn-5s and Br-4p orbitals. While conduction band minima, most of the part is contributed with the Sn-5p and 4 s, 4p orbitals of Br atom.

### 3.2 Strain engineering

**Fig. 5:**
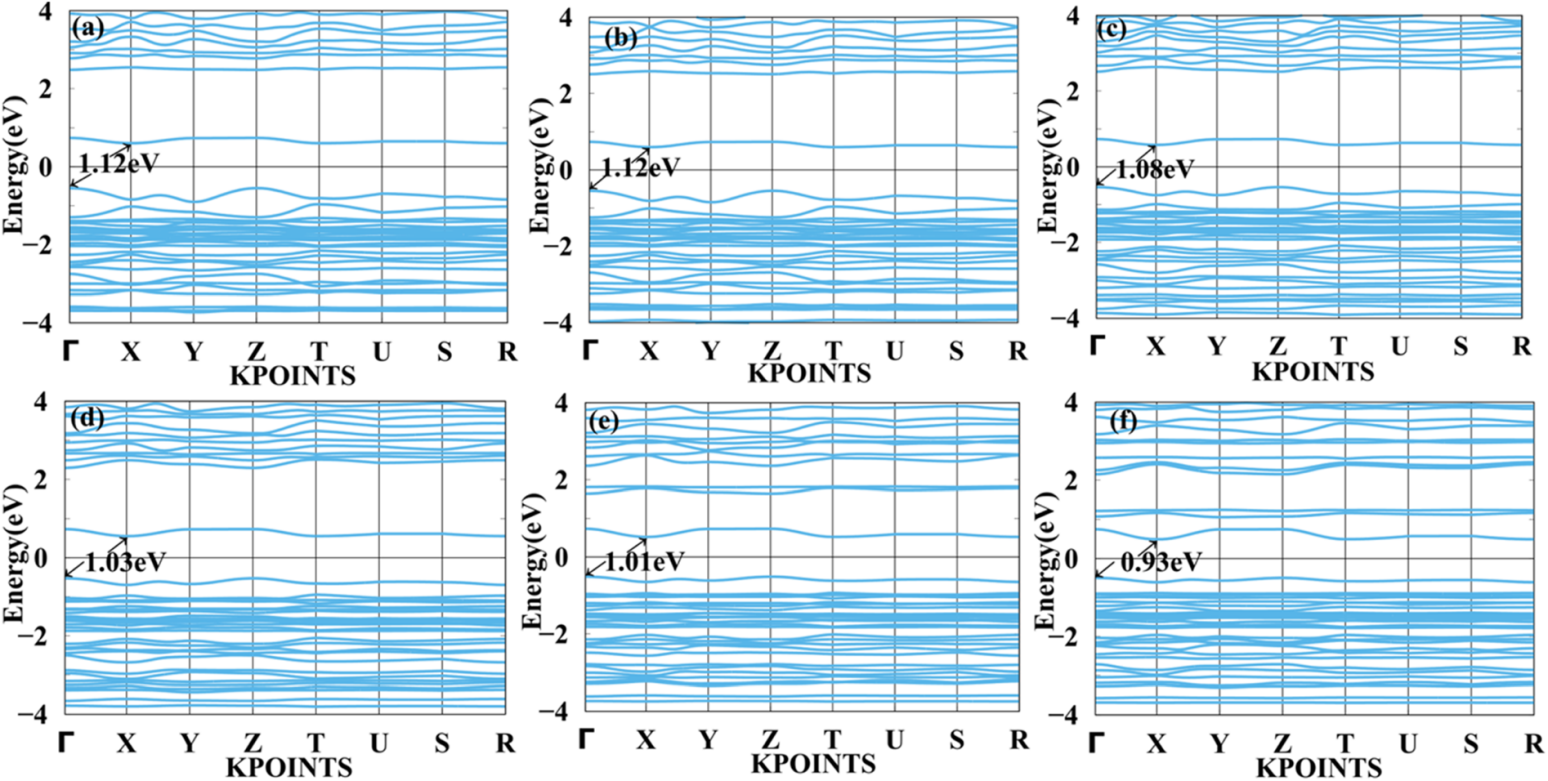
Calculated tensile strain band structure of MASnBr_3_ with (a) 1%, (b) 2%, (c) 4%, (d) 6%, (e) 8% and (f) 10% respectively.

**Fig. 6:**
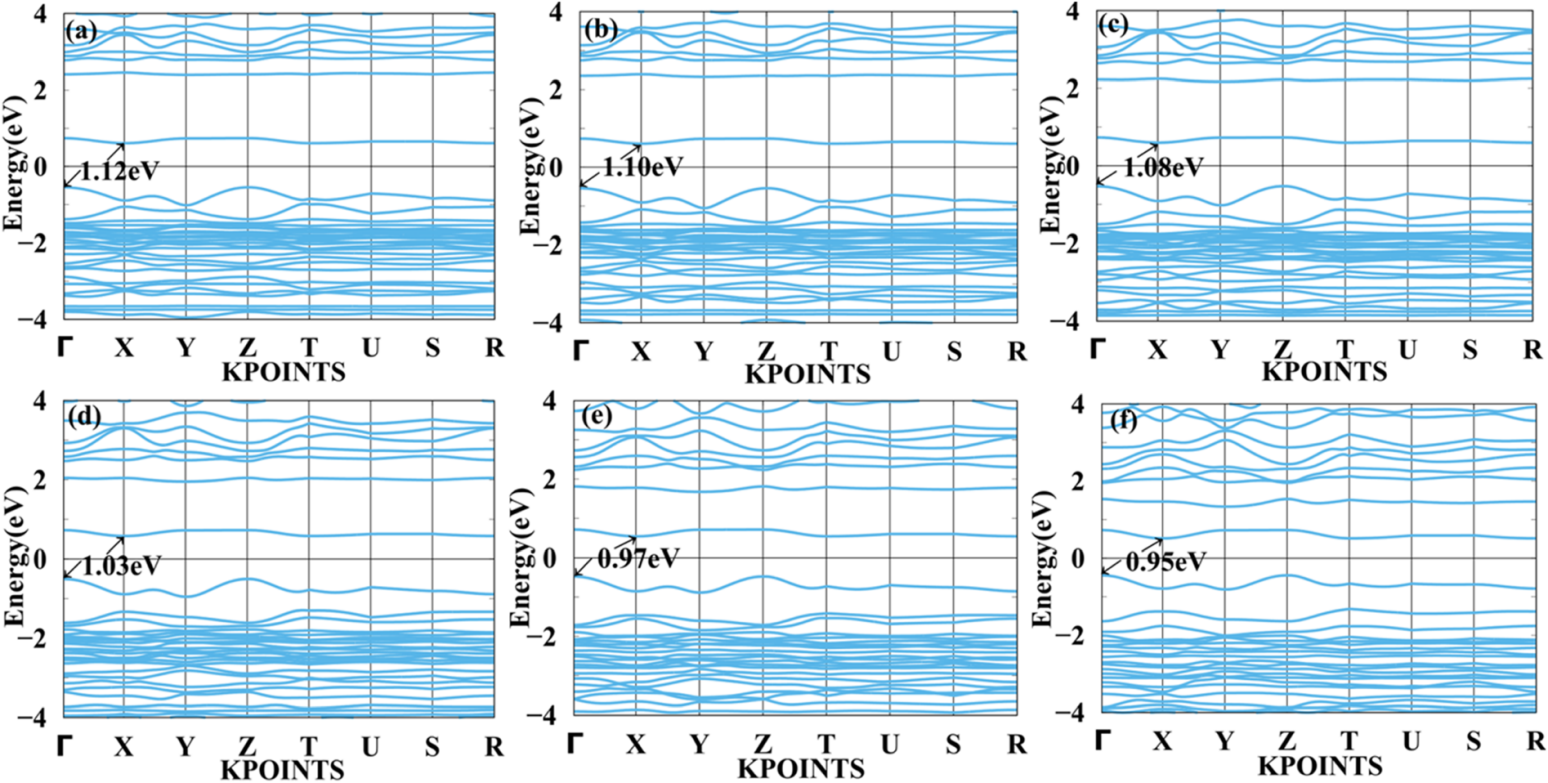
Calculated compressive strain band structure of MASnBr_3_ with (a) 1%, (b) 2%, (c) 4%, (d) 6%, (e) 8% and (f) 10% respectively.

**Fig. 7:**
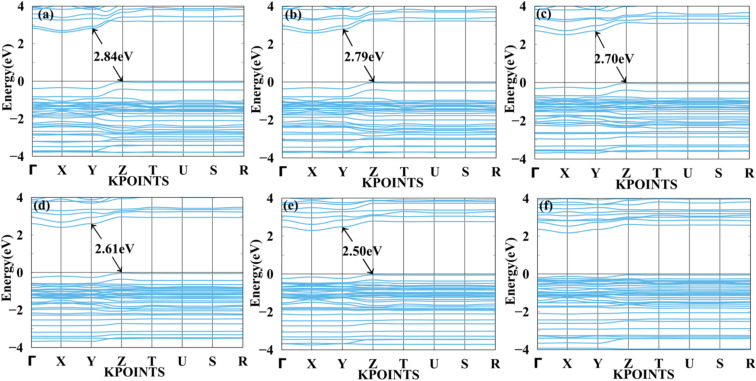
Calculated tensile strain band structure of BAMASn_2_Br_7_ with (a) 1%, (b) 2%, (c) 4%, (d) 6%, (e) 8% and (f) 10% respectively.

**Fig. 8:**
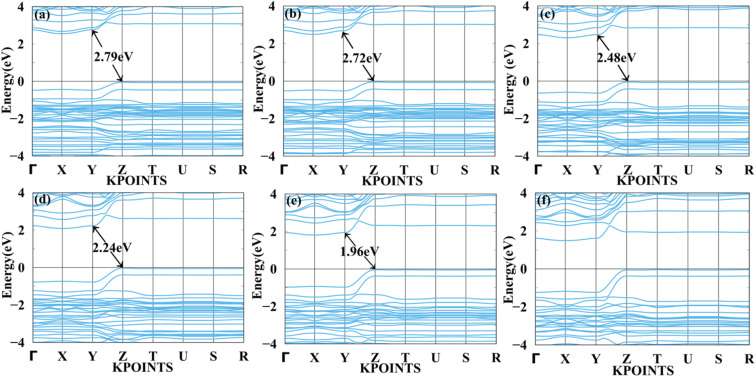
Calculated compressive strain band structure of BAMASn_2_Br_7_ with (a) 1%, (b) 2%, (c) 4%, (d) 6%, (e) 8% and (f) 10% respectively.

We applied mechanical strain to both systems to moderate electronic properties as shown in Fig. 5 and 6 for MASnBr_3_, Fig. 7 and 8 for BAMASn_2_Br_7_. Due to flexible structural and compositional properties, mechanical strain is an efficient way to tune electronic properties.^5,41^ As per literature survey, we found that there are many experimental as well as theoretical techniques have been developed to improve only performance of the system or device. In the literature survey we found that there are no other studies on strain dependent on 2D MASnBr_3_ and Ruddlesden–Popper BA_2_MASn_2_Br_7_ orthorhombic structures. Lattice strain is promising technique to improve performance of the many 2D materials. This has been useful for both experimental as well as theoretical groups. As per experimental researches studies of hetero epitaxial perovskite thin films shows that lattice strain play an important role to determining the physical properties of the thin films.^5,41,60,61^ In the case of the MASnBr_3_ system, we have applied both tensile and compressive strain along the *x* and *y* directions from 1% to 10% in the interval of 2%. Under tensile strain, condition bandgap was consistently decreased from 1% to 10% from 1.12 eV to 0.93 eV, as shown in Fig. 5. Similarly, compressive strain band gap decreases consistently 1% to 10% from 1.12 eV to 0.95 eV as shown in Fig. 6. Compared with the unstrained system with both tensile and compressive strain systems, the bandgap was decreased because the valence band contains most of the part of antibonding states of Br-p and Sn-s orbitals. In comparison, the conduction band contains most of the non-antibonding states of Br-p and Sn-p orbitals. Therefore near the Fermi level, downward shifting of conduction bands and upward shifting of valence band occurred effectively.

Fig. 7 and 8 show the tensile and compressive strain-dependent band structure of BAMASn_2_Br_7_ perovskites. Here, under conditions of tensile strain, bandgap reduces as strain is increased by 1%, 2%, 4%, 6%, or 8%, going from 2.84 eV to 2.792 eV to 2.70 eV to 2.61 eV to 2.50 eV. Similarly, in compressive strain conditions, band gaps decrease from 1% to 8% to 2.84 eV to 1.96 eV. At 10% both types of strain show metallic behaviour. That means the sustainability of the structure is up to 8%. Further, in Ruddlesden–Popper BAMASn_2_Br_7_. Comparison between the unstrained systems with strain systems of nature of the bandgap varies. Due to the presence of antibonding states of Br-p and Sn-s orbitals in valence bands. Non-antibonding states of Br-p and Sn-p orbital's in conduction bands are similar as that of the MASnBr_3_ perovskites. Overall, the mechanical strain technique is useful for tuning the bandgap and open the path for experimentalists to study thin films' physical and chemical properties.^5,60^

**Fig. 9:**
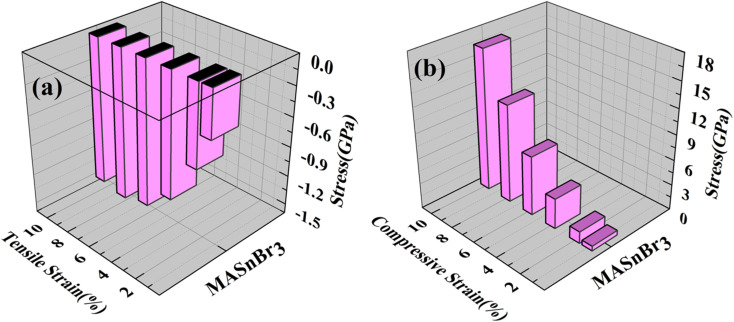
Applied (a) tensile and (b) compressive strain *versus* stress for the MASnBr_3_.

**Fig. 10:**
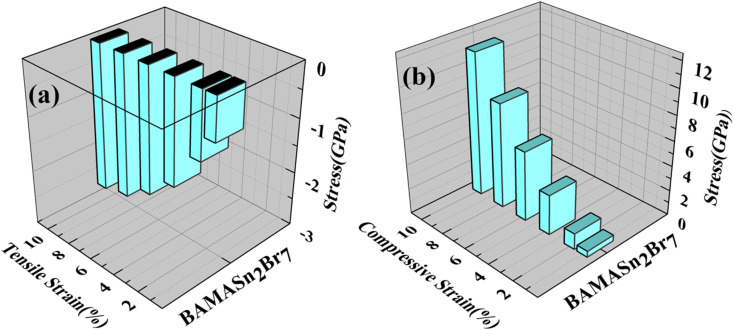
Applied (a) tensile and (b) compressive strain *versus* stress to the BAMASn_2_Br_7_.

Using the Hellmann–Feynman theorem^61^ In Fig. 9 and 10 we have calculated stress on both systems. In both cases, we applied strain on the overall system along the *x*-axis and *y*-axis for MASnBr_3_*x*-axis and *z*-axis for BAMASn_2_Br_7_. Overall stress was increased from 1% to 10%. Compared with tensile and compressive strain, in both systems, compressive stress is more and tensile stress is less. This change is happened due to contractions and expansion of lattice parameters of the system as shown in 2D barplot in Fig. S1.† Which effect the inter-bonding activity of the atoms. In the case of the MASnBr_3_ system, the structure's sustainability is up to 10% in both tensile and compressive. While in the case of BAMASn_2_Br_7_, the sustainability of the strain is up to 8%. After that, both systems show metallic nature.

### 3.3 Charge carrier mobility

Further, we have a focus on the charge transport properties of both systems. Due to flexible structural and compositional properties, these systems are well sustained in strain conditions, bands experience a high degree of dispersion and have a bandgap near optical band gaps.^51^ Near the Fermi level, the directional dependent effective mass of both electrons and holes with the slope of *x*, *y*, *z*-axis direction are shown in Table 2. The effective mass of the electron and holes for MASnBr_3_ along *x* and *y* directions are 0.36 to 0.057 and for Ruddlesden–Popper BAMASn_2_Br_7_ along *x* and *z*-direction are 0.33 to 0.146. In both cases, the effective mass of electrons and holes is remarkably more than the previously reported.^35,43,62^

**Table 2:** Calculated effective mass, carrier mobility, and relaxation time of MASnBr_3_ and BAMASn_2_Br_7_ respectively

System		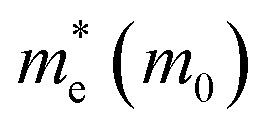	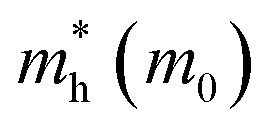	*C* (N m^−1^)	*μ*_e_ (cm^2^ V^−1^ s^−1^)	*μ*_h_ (cm^2^ V^−1^ s^−1^)	*τ*_e_ (fs)	*τ*_h_ (fs)
MASnBr_3_	*X*	0.122	0.0578	19.17	248.0	181.6	30.25	10.49
	*Y*	0.361	0.0633	19.17	404.2	800	146	50.64
BAMASn_2_Br_7_	*X*	0.160	0.336	7.97	557	124.1	89.34	41.69
	*Z*	0.146	0.320	7.97	367.4	779	53.64	249

We also calculate the charge transport carrier mobility and relaxation time using deformation potential theory. Which leads to calculate using formula2
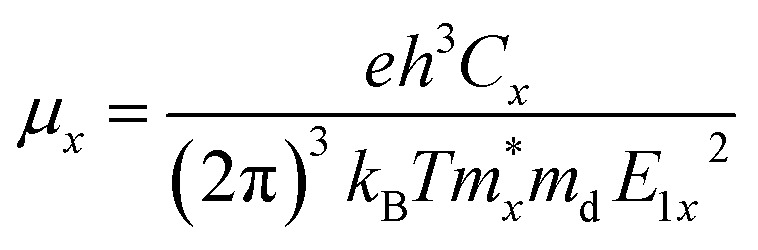


Also carrier mobility dependent relaxation time (*τ*), *τ* = (μm*)/*e* as shown in Table 2. Here *e* stand for electron charge, *h* for Planck's constant, m* for effective mass 
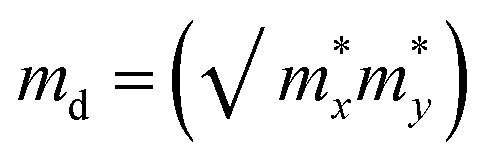
, *E*_1*x*_ = Δ*V*/(Δ*a*_*x*_/*a*_*x*_) deformation potentials.^5^ In the case of MASnBr_3_, the calculated carrier mobility of holes is higher than the electrons. Similar trends were observed in BAMASn_2_Br_7_, the hole carrier mobility is higher than the electrons. In compared with lead-iodide-based perovskites, carrier mobility of Sn-bromide-based perovskite is 14% and 24% higher, which are quite larger than the reported experimental and theoretical data.^5,35,41,43,62^ This anisotropic nature of carrier mobility is due to smaller effective mass and flexible structural properties, affecting the band gaps. This high carrier mobility leads to better device performance. Open a new path for experimentalists to find solar cell efficiency and developed optoelectronic devices.

### 3.4 Optical properties

**Fig. 11:**
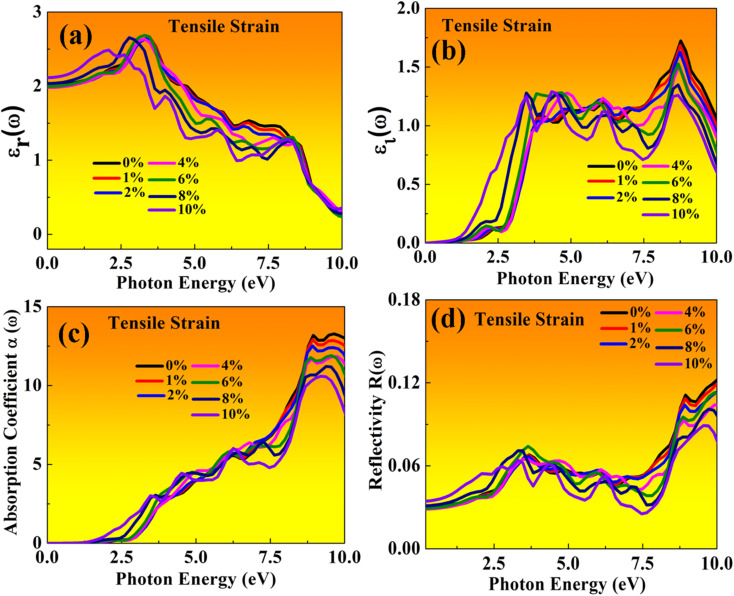
Optical properties of MASnBr_3_ perovskites represented with tensile strain: (a) real *ε*_r_(*ω*), (b) imaginary *ε*_i_(*ω*), (c) absorption coefficient, (d) reflectivity.

**Fig. 12:**
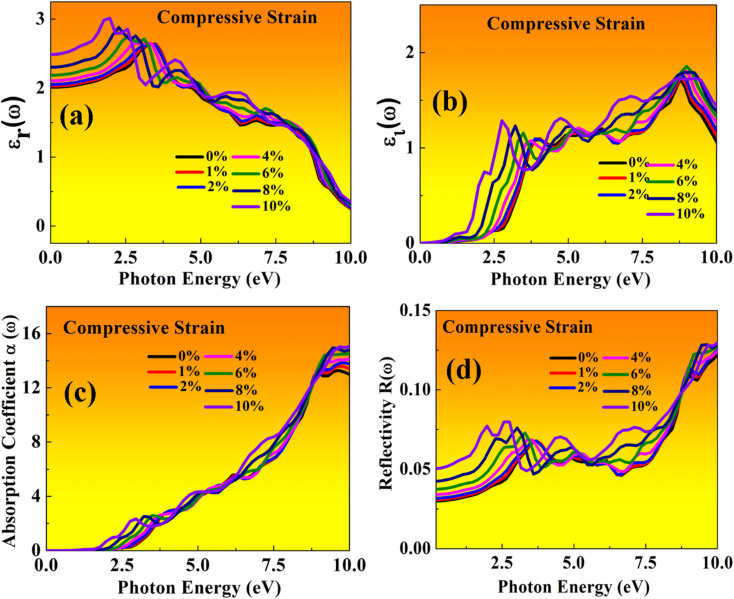
Optical properties of MASnBr_3_ perovskites represented with compressive strain: (a) real *ε*_r_(*ω*), (b) imaginary *ε*_i_(*ω*), (c) absorption coefficient, (d) reflectivity.

**Fig. 13:**
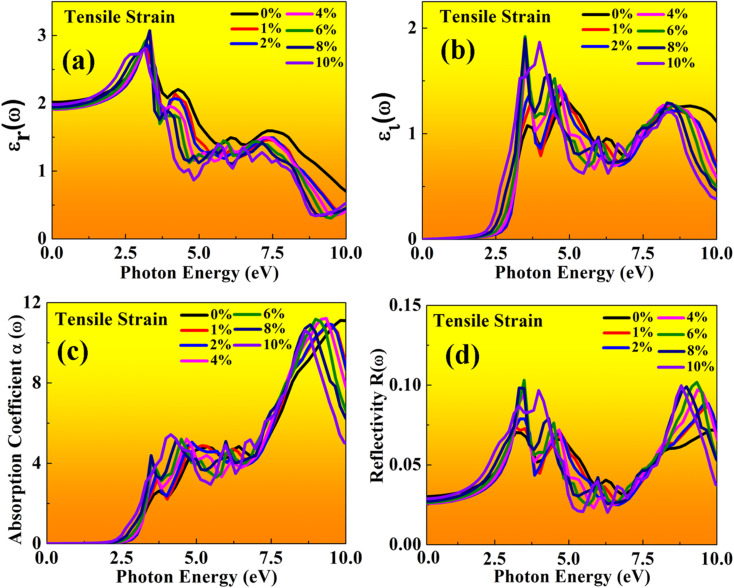
Optical properties of BAMASn_2_Br_7_ perovskites represented with tensile strain: (a) real *ε*_r_(*ω*), (b) imaginary *ε*_i_(*ω*), (c) absorption coefficient, (d) reflectivity.

**Fig. 14:**
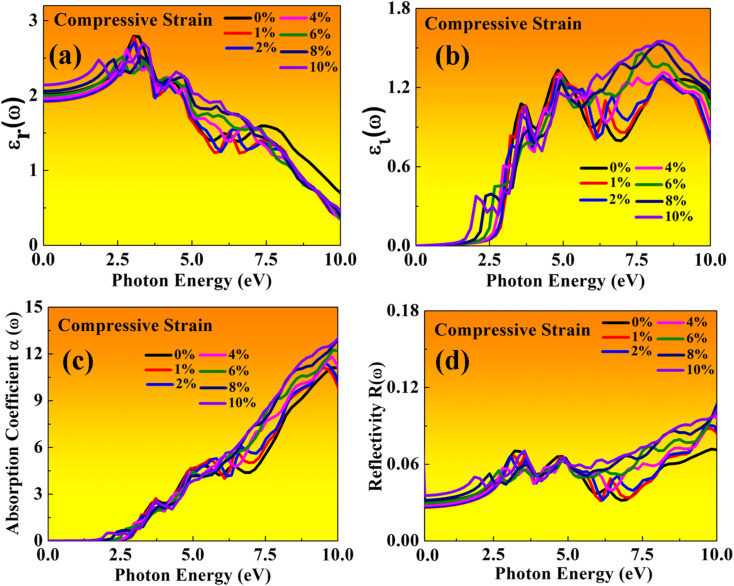
Optical properties of BAMASn_2_Br_7_ perovskites represented with compressive strain: (a) real *ε*_r_(*ω*), (b) imaginary *ε*_i_(*ω*), (c) absorption coefficient, (d) reflectivity.

Here, both perovskites structures have flexible electronic and structural properties, which is useful for determining their optical behaviour in both tensile and compressive strains. Using frequency-dependent Kramer–Kronig relation developed by Drude model.^70,71^ We are focused on optical properties in Fig. 11 and 12 shown in MASnBr_3_ under tensile and compressive strains, while in Fig. 13 and 14 shows optical properties of BAMASn_2_Br_7_ under mechanical strain conditions.

In the real part of the MASnBr_3_, the static dielectric constant is 2.011. Further, increases in tensile strain the static dielectric constant from 1.99 to 2.11. In compressive strain from 2.02 to 2.48 for 1% to 10%, as shown in Fig. 11(a) and 12(a), respectively. In the case Ruddlesden–Popper BAMASn_2_Br_7_ optical properties, the real part static dielectric constant is 2.01. Under mechanical strain conditions, the static dielectric constant decreases in tensile strain from 2.01 to 1.98 from 1% to 10%, while in the case of compressive strain static dielectric constant are increases from 1.9 to 2.14 from 1% to 10% as shown in Fig. 13(a) and 14(a) respectively. These high values of static dielectric constant are useful to define optoelectronic device performance. Similarly, in both cases, the imaginary part of static dielectric constant the optical activity is due to the transition of the electron in valence band Sn-s states to Br-p states in the conduction band.

In both cases, the first peak is at 2.5 eV. After applying both tensile and compressive strain, optical activity orientation is a move towards high energy part 8 eV as shown in Fig. 11(b) and 12(b) for MASnBr_3_, Fig. 13(b), 14(b) for BAMASn_2_Br_7_. Further, optical properties are analyzed by absorption coefficient, reflectivity. In the case of MASnBr_3_ absorption coefficient is at 13.27 × 10^5^ cm^−1^ at 8.8 eV. Which are further decreases from 12.91 × 10^5^ cm^−1^ to 10.58 × 10^5^ cm^−1^ in tensile strain from 1% to 10% as shown in Fig. 11(c). In case compressive strain absorption coefficient increases from 13.60 × 10^5^ cm^−1^ to 15.25 × 10^5^ cm^−1^ for 1% to 10% as shown in Fig. 12(c). Also, in the case of BAMASn_2_Br_7_ absorption coefficient is 11.11 × 10^5^ cm^−1^ for the unstrained system. For tensile strain it is decreases from 10.91 × 10^5^ cm^−1^ to 10.62 × 10^5^ cm^−1^ for 1% to 10% as shown in Fig. 13(c) and in case of compressive strain it is increases from 11.14 × 10^5^ cm^−1^ to 13.38 × 10^5^ cm^−1^ for 1% to 10% as shown in Fig. 14(c). The first small peak orientation is near 2.5 eV, which shows optical band gap activity is in the range of previously reported data.^33,35,47^ Overall, the optical activity of MASnBr_3_ and BAMASn_2_Br_7_ are shown. By reflectivity by the orientation of peaks at 0 eV to 2.5 eV and 2.5 eV to 5 eV, and further 10 eV is in the visible (500–414 nm) as well as ultraviolet region(310–177 nm) of the energy spectrum as shown if Fig. 11(d), 12(d), 13(d) and 14(d). Therefore maximum light reflects in the visible as well as in the ultraviolet region. These studies are consistent with the previously reported experimental and theoretical studies.^33,35,47^ Overall, compared with MASnBr_3_ and BAMASn_2_Br_7_ perovskites with other 2D hybrid halide perovskites. Tunable band gap and optical, charge transport activity useful for photovoltaic and solar cell devices.

### 3.5 Solar cell efficiency parameter

Further, we have investigated the solar cell efficiency performance of 2D MASnBr_3_. The Shockley and Queisser (SQ) model depend on the material's bandgap. To calculate the performance of solar cell efficiency, the band gap are required to be in between 1.0 to 1.7 eV. Therefore, the bandgap of 2D MASnBr_3_ is under SQ limit and band gap of BAMASn_2_Br_7_ are higher than the SQ limit. The solar cell parameters like open-circuit voltage (*V*_oc_), fill factor (FF), short circuit current density (*J*_sc_), and solar cell efficiency are calculated as per previously reported results.^5,41^ More information is provided in the ESI† as shown in Tables 3, S1 and S2.†

**Table 3:** Represents calculated solar cell parameters for open circuit voltage, fill factor, short circuit current density, power conversion efficiency of MASnBr_3_ monolayer

	*V*_oc_ (eV)	FF	*J*_sc_ (mA cm^−2^)	*H* (%)
MASnBr_3_	2.64	0.94	9.40	23.46

For 2D MASnBr_3_ solar cell efficiency is 23.46%. Which is 18% larger than that of the previously reported lead-based 2D MAPbI_3_ (ref. 43) perovskites. Also, we have found that in comparison with experimental results. The power conversion efficiency of lead-free perovskites developed by Hao *et al.* and Xu *et al.* group have reached up to 5.73% and 8.79%, respectively.^49,51^ Overall in comparison with lead-free 3D hybrid halide perovskites with 2D hybrid halide perovskites. The 2D hybrid halide perovskites are more efficient and suitable candidates to develop new generation solar cells.^5,41,43,49,51,57^ Under both tensile and compressive strain conditions. The calculated solar cell efficiency varies from 13% to 23% as shown in Tables 3, S1 and S2.† Therefore we conclude that the lead-free 2D MASnBr_3_ is a suitable candidate for solar cell and photovoltaic applications.

## 4. Conclusions

Here, the 2D MASnBr_3_ and Ruddlesden–Popper perovskites BAMASn_2_Br_7_ hybrid halide perovskites have flexible structural and compositional properties. Also, the calculated bandgap and lattice parameters of MASnBr_3_ and Ruddlesden–Popper perovskites BAMASn_2_Br_7_ are consistent with previously reported experimental and theoretical results. Under mechanical strain, *i.e.*, tensile and compressive strain tunable bandgap activity is consistent with previously reported, showing sustainability of the structure up 10% in MASnBr_3_ and up to 8% in BAMASn_2_Br_7_. We found highest carrier mobility for electron up to 404 cm^2^ V^−1^ s^−1^ and holes 800 cm^2^ V^−1^ s^−1^ for MASnBr_3_, and BAMASn_2_Br_7_ highest carrier mobility up to 557 cm^2^ V^−1^ s^−1^ for electron and up to 779 cm^2^ V^−1^ s^−1^ for the hole, which are 14% and 24% higher than the reported data. The calculated solar cell parameters open-circuit voltage (*V*_oc_), fill factor (FF), short circuit current density (*J*_sc_), and solar cell efficiency are consistent with the previously reported experimental and theoretical results. Here, tin-based 2D MASnBr_3_ shows high efficiency up to 23.46%, 18% higher than the lead-based perovskites. In optical activity, a high static dielectric constant shows good device performance. With high absorption coefficient up to 15.25 × 10^5^ cm^−1^ for MASnBr_3_ and BAMASn_2_Br_7_ absorption coefficient up to 13.38 × 10^5^ cm^−1^. Therefore overall results of this theoretical study. With mechanical strain engineering may be convenient to the experimentalist support to improve the performance of the 2D perovskites. This study supports these materials as promising candidates for photovoltaic and solar cell device applications.

## Conflicts of interest

There are no conflicts to declare.

## Supplementary Material

RA-013-D3RA00108C-s001
