# Supplementary material for: Lead-free 2D MASnBr3 and Ruddlesden–Popper BA2MASn2Br7 as light harvesting materials
Source: RSC Adv. 2023 Mar 9;13(12):7939–51. doi: 10.1039/d3ra00108c (PMC9997451; doi:10.1039/d3ra00108c)
Supplement: RA-013-D3RA00108C-s001 [file RA-013-D3RA00108C-s001.pdf]

Electronic Supplementary Material (ESI) for RSC Advances.
This journal is © The Royal Society of Chemistry 2023

Supplementary Information

Lead-Free 2D $MASnBr_3$ and Ruddlesden-Popper $BA_2MASn_2Br_7$ as Light Harvest Materials

Sandip R. Kumavat[a], Yogesh Sonvane[a*]

Advanced Materials Lab, Department of Physics, Sardar Vallabhbhai National Institute of Technology, Surat 395007, India

Solar Cell Efficiency Parameter

Moreover, we have investigated that 2D $MASnBr_3$ perovskite materials have strong optical absorption which is useful to improve solar cell efficiency. To find the performance of the 2D $MASnBr_3$ layer structure in the solar cell device. To better understand the performance of the solar cell, we have calculated open-circuit voltage (Voc), fill factor (FF), power conversion efficiency (η), short circuit current density (Jsc). As we know, Shockley and Quessier (SQ) model depend on the band gap (E_g) of material to calculate the performance of solar cell efficiency which gives A(E) = 1 if $E>E_g$ and A(E) = 0 for $E< E_g$ [5,41]. With all this calculation of solar cells, parameters are good agreement with the previously reported in experimental as well as theoretical results [5,41,43].

Table S1: Represents calculated solar cell parameters for tensile strain open circuit voltage, Fill Factor, short circuit current density, power conversion efficiency of of $MASnBr_3$ monolayer.

$MASnBr_3$	**0 %**	**2 %**	**4 %**	**6 %**	**8 %**	**10%**
**V_{oc} (eV)**	2.64	2.64	2.68	2.70	2.71	2.75
**FF**	0.9453	0.9453	0.9459	0.9462	0.9465	0.9471
**Jsc (mA/cm^2)**	9.40	9.03	8.35	7.31	6.94	5.56
**η(%)**	23.46	22.54	21.43	18.64	17.83	14.83

Table S2: Represents calculated solar cell parameters for compressive strain open circuit voltage, Fill Factor, short circuit current density, power conversion efficiency of of $MASnBr_3$ monolayer.

$MASnBr_3$	**0 %**	**2 %**	**4 %**	**6 %**	**8 %**	**10%**
**V_{oc} (eV)**	2.64	2.64	2.66	2.68	2.71	2.75
**FF**	0.9453	0.9453	0.9456	0.9459	0.9465	0.9471
**Jsc (mA/cm^2)**	9.40	8.64	8.25	7.31	6.22	5.07
**η(%)**	23.46	21.56	21.74	18.51	15.98	13.21

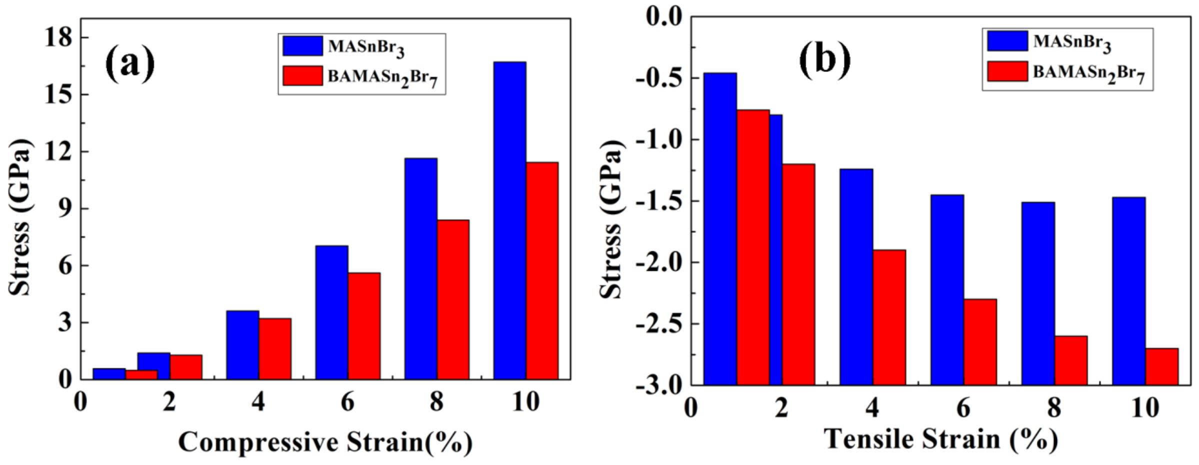

Fig. S1. Applied (a) Compressive and (b) Tensile strain varses Stress of $MASnBr_3$ and $BAMASn_2Br_7$.
